# Distance to home does not influence treatment success during and after inpatient treatment in adolescents with anorexia nervosa

**DOI:** 10.1007/s00787-022-02013-7

**Published:** 2022-06-08

**Authors:** Adrian Meule, David R. Kolar, Silke Naab, Ulrich Voderholzer

**Affiliations:** 1 Department of Psychiatry and Psychotherapy, University Hospital, LMU Munich, Munich, Germany; 2 Schoen Clinic Roseneck, Am Roseneck 6, 83209 Prien am Chiemsee, Germany; 3 Medical Center, Department of Psychiatry and Psychotherapy, Faculty of Medicine, University of Freiburg, Freiburg, Germany

**Keywords:** Anorexia nervosa, Adolescents, Inpatient treatment, Distance to home, Treatment outcome

## Abstract

Current treatment guidelines recommend that inpatients with eating disorders—particularly adolescents with anorexia nervosa—should receive treatment at facilities within close distance to their home. However, whether distance to home actually influences short- and long-term treatment outcome in adolescents with anorexia nervosa has not been investigated yet. We re-analyzed data at admission, discharge, and 1-year follow up from a recent study with *N* = 142 female, adolescent inpatients with anorexia nervosa. Distance to home did not moderate changes in body weight, eating disorder symptoms, depressive symptoms, compulsive exercise, and life satisfaction. This is the first analysis that indicates that specialized inpatient treatment for adolescents with anorexia nervosa is effective both close to and away from home.

## Introduction

Current treatment guidelines (e.g., by the National Institute for Health and Care Excellence and the American Psychiatric Association) recommend that inpatients with eating disorders—particularly adolescents with anorexia nervosa (AN)—should receive treatment at facilities within close distance to their home [1, 2]. Being an inpatient may carry with it a pervasive sense of being removed from the outside world and the normality of adolescent life [3], which may be exacerbated when the treatment facility is located far from home. Other considerations for preferring facilities close to home include caregiver costs such as travel expenses [4]. However, empirical evidence for preferring inpatient treatment close to home is virtually non-existent. For example, longer distance to home related to longer length of stay in inpatients with AN in one study [5] but was unrelated to length of stay in another [6]. Furthermore, it seems that the influence of distance to home on actual treatment success during and after inpatient treatment has not been examined yet.

## Methods

We re-analyzed data from a recently published study that investigated changes in body weight, eating disorder symptoms and other variables during and after inpatient treatment in adolescents with AN across admission, discharge, and one-year follow up. The study was approved by the institutional review board of the LMU Munich. One-hundred and forty-two female adolescents with AN who received inpatient treatment at the Schoen Clinic Roseneck (Prien am Chiemsee, Germany) between 2016 and 2018 were included in this study, 121 of which participated at follow up. Dependent variables were age- and sex-specific body mass index (BMI) percentiles and total scores of the Eating Disorder Examination–Questionnaire, Beck Depression Inventory–II, Commitment to Exercise Scale, and Satisfaction With Life Scale. A detailed description of the sample, treatment elements, and measures can be found in the article by Meule et al. [7], which is openly accessible.

The Schoen Clinic Roseneck treats patients that reside all over Germany and Austria, that is, there is a large diversity in terms of distance to home. Treatment of adolescents includes family therapy sessions both in person and by phone or video calls. Patients are allowed to receive visitors, ideally on weekends. If appropriate for the current treatment stage, patients are also allowed to leave the hospital at weekends, including home visits overnight that are previewed before and reviewed afterwards with the patients’ therapists. These treatment elements are integrated depending on the current treatment stage and the patients’ physical and mental conditions but irrespective of distance to home. If a long distance to home poses a challenge, alternative solutions are sought (e.g., instead of traveling home, parents may come to visit and the patient is allowed an overnight stay at the hotel). The hospital does not offer subsequent outpatient treatment after completion of inpatient treatment but aims to organize outpatient treatment with a local practitioner at or near the patients’ place of residence. Ideally, patients should receive their outpatient treatment from the therapist who provided therapy before the inpatient stay.

**Fig. 1:**
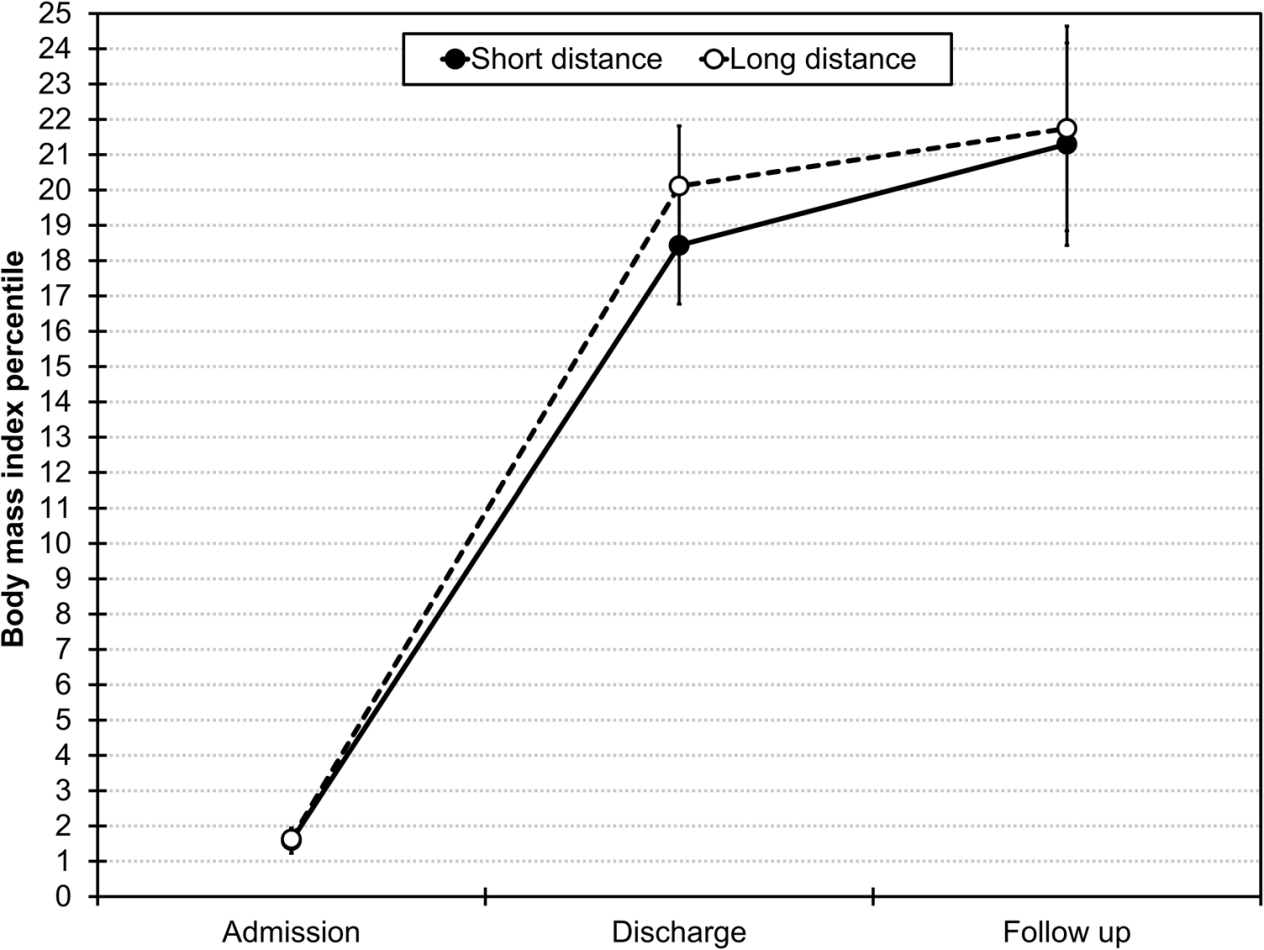
Body mass index percentiles across measurements as a function of short (< 239.90 km) and long (> 239.90 km) distance to home. Error bars represent the standard error of the mean

In the original report [7], data were analyzed with mixed-effects models [8]. This analytic strategy has multiple advantages as compared to, for example, analysis of variance. For instance, it can handle missing data better (i.e., cases with missing data are not excluded), both categorical and continuous predictor variables can be used, random effects can be specified, and both linear and non-linear trajectories can be modeled. Compulsive exercise and life satisfaction changed linearly across the three measurements, which is why these variables were predicted by a linear time term only. Changes in BMI percentiles, eating disorder symptoms, and depressive symptoms changed non-linearly across the three measurements (cf. Figure 1), which is why a second-order orthogonal polynomial of the time term (i.e., time^2^) was added as predictor variable. The models also included random intercepts of patients. For the current analyses, we determined the linear distance between the hospital and each patient’s place of residence with Google Maps (https://www.google.com/maps). To test whether distance to home moderated changes across the three measurements, we added the fixed effect of distance to home on all time terms. For these analyses, we report unstandardized regression coefficients, standard errors, and *p* values for the highest-order interactions. As there were five dependent variables (i.e., five models were calculated), we set the level of significance to 0.05/5 = 0.01. At this level, the minimal detectable unstandardized regression coefficient with more than 80% power was 0.025 for the time^2^ × distance interaction. This corresponds to a detectable difference of 2.44 BMI percentile points due to this interaction for an individual with a distance of 238.67 km (mean distance across participants) at follow up, compared to an individual living in close vicinity of the treatment center. The data and code for all analyses can be accessed at https://osf.io/qvmwr.

## Results

Distance to home ranged between 4.13 and 697.49 km (*M* = 238.67, *SD* = 153.49, *SE* = 12.88, percentiles: 25th = 104.76, 50th = 239.90, 75th = 329.58) and was unrelated to length of stay (*r* = 0.042, *p* = 0.621). The interaction of the quadratic time term and distance to home (i.e., time^2^ × distance) for predicting changes in BMI percentiles was not significant (estimate = − 0.0002, *SE* = 0.01, *p* = 0.977). As is depicted in Fig. 1, changes in BMI percentiles across the three measurements were similar for patients with a long and short distance to home (based on a median split). Note, however, that this graphical depiction merely serves the purpose of visualizing the non-significant interaction effect time^2^ × distance, that is, distance to home was used as a continuous variable in all models and was not categorized into groups. The interaction time^2^ × distance was also not significant when examining changes in eating disorder symptoms (estimate = 0.0003, *SE* = 0.001, *p* = 0.690) and depressive symptoms (estimate = 0.01, *SE* = 0.01, *p* = 0.168). Finally, the interaction between the linear time term and distance to home (time × distance) was also not significant when examining changes in compulsive exercise (estimate = 0.001, *SE* = 0.0003, *p* = 0.012) and life satisfaction (estimate = − 0.004, *SE* = 0.003, *p* = 0.206).

## Discussion

In the current study, distance to home did not moderate treatment success during and after inpatient treatment in adolescents with AN. This finding resonates with reports from Germany that examined patients with depression [9] and substance use disorders [10], in which distance to home did not influence treatment success either. An auxiliary finding was that distance to home was unrelated to length of stay, thus contrasting results reported by Maguire et al. [5] but corroborating those by Strik Lievers et al. [6]. Yet, studies that replicate our findings—particularly outside of Germany—are urgently needed. For example, 94% of patients who participated at follow up in the current study indicated that they received outpatient psychotherapy after discharge (cf. [7]). This may have attenuated any influence of distance to home as psychotherapeutic aftercare was provided for almost all patients irrespective of their place of residence, which may not be the case in countries with other healthcare systems. Yet, while the current data were collected before the COVID pandemic, we expect that remote post-inpatient aftercare interventions (e.g., delivered via videoconference [11]) will increase in the years to come, which may further reduce the importance of distance to home on treatment outcome after discharge as provision of psychotherapeutic aftercare by the hospital is no longer dependent on place of residence.

Strengths of the current study include the large sample size, repeated measurements, and large range of distance to home, which allowed us to test meaningful effects that distance to home might have on short- and long-term treatment success in adolescents with AN. As this was a re-analysis of existing data, however, we could not take important variables into account that may influence effects of distance to home on treatment outcome. These may include health economic variables (e.g., loss of earning for family members secondary to increased travel time or travel expenses) and social factors (e.g., not being able to attend school or work or see family and friends when on leave from the inpatient unit). As effects of distance to home on treatment outcome appears to be such an under-researched topic, future studies may also consider applying qualitative research designs to gain further insights into the experiences by both patients and their relatives during and after treatment.

Both inpatient treatment far away from and close to home have advantages and disadvantages. For example, advantages of inpatient treatment far away from home include keeping distance to a potentially harmful social environment and having more discretion during treatment. Moreover, German treatment guidelines recommend that inpatient treatment of AN should take place in facilities that offer a specialized, multimodal treatment program [2], which may not be feasible if there is no such specialized facility near patients’ homes. In contrast, centers with both in- and outpatient units can ensure continuity from inpatient to outpatient treatment by the same practitioners, which is not feasible when patients receive inpatient treatment far from home and need to return after inpatient treatment. As of yet, however, there have been no studies examining whether distance to home actually influences short- and long-term treatment outcome in adolescents with AN. This analysis is the first to indicate that specialized inpatient treatment for adolescents with AN is effective both close to and away from home.
